# Rational design of potent ultrashort antimicrobial peptides with programmable assembly into nanostructured hydrogels

**DOI:** 10.3389/fchem.2022.1009468

**Published:** 2023-01-13

**Authors:** Priscila Cardoso, Samuel Appiah Danso, Andrew Hung, Chaitali Dekiwadia, Nimish Pradhan, Jamie Strachan, Brody McDonald, Kate Firipis, Jacinta F. White, Arturo Aburto-Medina, Charlotte E. Conn, Céline Valéry

**Affiliations:** ^1^ School of Health and Biomedical Sciences, Translational Immunology and Nanotechnology Theme, NanoBioPharm Research Group, RMIT University, Bundoora, VIC, Australia; ^2^ School of Science, STEM College, RMIT University, Melbourne, VIC, Australia; ^3^ Materials Characterisation and Modelling, Manufacturing, CSIRO, Clayton, VIC, Australia; ^4^ RMIT Microscopy and Microanalysis Facility (RMMF), RMIT University, Melbourne, VIC, Australia; ^5^ BioFab3D, Aikenhead Centre for Medical Discovery, St Vincent’s Hospital Melbourne, Fitzroy, VIC, Australia; ^6^ Biomedical and Electrical Engineering, School of Engineering, RMIT University, Melbourne, VIC, Australia

**Keywords:** antimicrobial peptide (AMP), peptide hydrogels, molecular engineering, molecular self assembly, fmoc-peptides

## Abstract

Microbial resistance to common antibiotics is threatening to cause the next pandemic crisis. In this context, antimicrobial peptides (AMPs) are receiving increased attention as an alternative approach to the traditional small molecule antibiotics. Here, we report the bi-functional rational design of Fmoc-peptides as both antimicrobial and hydrogelator substances. The tetrapeptide Fmoc-WWRR-NH_2_—termed Priscilicidin—was rationally designed for antimicrobial activity and molecular self-assembly into nanostructured hydrogels. Molecular dynamics simulations predicted Priscilicidin to assemble in water into small oligomers and nanofibrils, through a balance of aromatic stacking, amphiphilicity and electrostatic repulsion. Antimicrobial activity prediction databases supported a strong antimicrobial motif *via* sequence analogy. Experimentally, this ultrashort sequence showed a remarkable hydrogel forming capacity, combined to a potent antibacterial and antifungal activity, including against multidrug resistant strains. Using a set of biophysical and microbiology techniques, the peptide was shown to self-assemble into viscoelastic hydrogels, as a result of assembly into nanostructured hexagonal mesophases. To further test the molecular design approach, the Priscilicidin sequence was modified to include a proline turn—Fmoc-WPWRR-NH_2_, termed P-Priscilicidin–expected to disrupt the supramolecular assembly into nanofibrils, while predicted to retain antimicrobial activity. Experiments showed P-Priscilicidin self-assembly to be effectively hindered by the presence of a proline turn, resulting in liquid samples of low viscosity. However, assembly into small oligomers and nanofibril precursors were evidenced. Our results augur well for fast, adaptable, and cost-efficient antimicrobial peptide design with programmable physicochemical properties.

## Introduction

Microbial resistance to common antibiotics is threatening to cause the next pandemic crisis (WHO, 2020). In this context, antimicrobial peptides (AMPs) are receiving increased attention as an alternative approach to the traditional small molecule antibiotics (WHO, 2020).

Indeed, peptides are relatively easy to synthetise while exhibiting broad-spectrum antimicrobial activity, decreased microbial resistance and intrinsic biocompatibility. The emergence of molecular engineering principles to design antimicrobial peptide sequences has made rational sequence design a promising approach for rapid and adaptive design of novel antimicrobial therapeutics. Recent works demonstrated that sequences as short as 2–5 natural residues can display antimicrobial activity (Glossop et al., 2019; Cardoso et al., 2021). In parallel, a few natural and synthetic AMPs were reported to self-assemble in solution into nanostructures, a process that can be harnessed for drug delivery and material design (Cardoso et al., 2021).

Here we report the bi-functional rational design of Fmoc-tetrapeptides for potent antimicrobial activity and control of peptide hydrogelation through molecular self-assembly. Peptide molecular self-assembly is mediated through intermolecular non-covalent interactions such as hydrogen bonding, hydrophobic, ionic, and π−π stacking interactions (Cardoso et al., 2021). Pioneering works in this domain established that two nanostructure-forming short synthetic sequences - the diphenylalanine peptide and cyclic D,L-alpha alanine peptide—interact with bacterial membranes and display antibacterial activity (Fernandez-Lopez et al., 2001; Schnaider et al., 2017). The antimicrobial action of AMPs is known to mainly arise from a combination of electrostatic and hydrophobic interactions with microbial membrane components. Sequence motifs that favor antimicrobial activity have been reported, especially: i) presence of cationic amino acids ii) primary structure characterized by a net positive charge of 2–9, iii) presence of aromatic amino acids, iv) amphipathic character of primary sequence (Tian et al., 2015; Li et al., 2017; Cardoso et al., 2021).

Although there is still debate on a direct relationship between self-assembly and antimicrobial activity, networks of peptide self-assembled nanostructures present the advantage of potentially resulting into hydrogels through reticulation of the aqueous media. Such supramolecular hydrogels can be exploited to create biomaterials with various applications, including as drug delivery vehicles, tissue engineering scaffolds, or as precursors for creating nanostructured surfaces (Das and Gavel, 2020; Cardoso et al., 2021; Shi et al., 2021). Specifically, the use of a fluorenylmethyloxycarbonyl (Fmoc) group located at the N-terminal of the sequence was previously reported to drive peptide supramolecular assembly into biocompatible hydrogels (Fleming et al., 2013; Tao et al., 2016; Firipis et al., 2021).

By applying a bottom-up approach supported by molecular dynamics and online antimicrobial prediction tools detailed below, we designed two Fmoc-peptide sequences of four to five amino-acids. Both sequences entail motifs for antimicrobial and self-assembling, as detailed in the results section. Our rational design predicted both peptides to exhibit antimicrobial activity; however, with one sole hydrogelator sequence. Using a large set of biophysical and microbiological techniques, we investigated for both designed sequences: i) the self-assembly into different molecular species; ii) the kinetics and mechanisms of hydrogelation and iii) antimicrobial activity on different strains.

## Materials and methods

### Chemicals and reagents

Indolicidin (sequence: ILPWKWPWWPWRR-NH_2_, molecular weight: 1,906.3 g/mol) and FMOC-WWRR-NH_2_ (molecular weight: 924.1) were obtained from ChemPep (USA) as lyophilized powder and acetate salts with a purity >95%. Ethanol and Congo red were purchased from Sigma-Aldrich (Australia). Sodium chloride was purchased from Alfa Aesar (United Kingdom). The negative staining agent phosphotungstic acid was purchased from Proscitech (Australia). A Milipore filter system was utilized to obtain Milli-Q water. BD Difco Mueller Hinton Broth (MHB) and Sabouraud broth (SB) were purchased from Cell biosciences (Australia). Strains: *E. coli* NCTC 10418, *P. aeruginosa* ATCC 9721, *E. faecalis* ATCC 19433, *S. aureus* ATCC 25923, *S. aureus* ATCC BAA-1756 (methicillin resistant), *Streptococcus pneumoniae* ATCC 49619, *Candida albicans* ATCC 10231 and *Candida auris* DSM 21092 (Fluconazole resistant) were provided by the RMIT microbial culture collection. Bacterial strains were stored in 50% glycerol at −20 and −80°C.

### Peptide samples

The peptides were prepared by mixing the lyophilized peptide directly with type 1 water, vortexed for a few seconds to obtain an isotropic solution and stored at 4°C–5°C in between experiments.

### Molecular dynamics

The Fmoc-WWRR-NH_2_ and Fmoc-WPWRR-NH_2_ peptide structures were prepared using Swiss-PdbViewer (Deepview) (Guex and Peitsch, 1997) and Discovery Studio Visualizer (BIOVIA, Dassault Systèmes, Discovery Studio Visualizer, 2021 San Diego, CA). Molecular dynamics simulation cells for modelling peptide self-assembly were constructed by arranging smaller cubic ‘subcells’ containing a single peptide into larger cubic simulation cells composed of 3 × 3 × 3 subcells, in a crystalline structure, resulting in final cell sizes of 9 × 9 × 9 nm^3^ (WWRR) or 9.6 × 9.6 × 9.6 nm^3^ (WPWRR). Each of the simulation cells therefore contains 27 of the respective peptides initially in random orientations. Each box was filled with 23,054 (WWRR) or 28,273 (WPWRR) TIP4P water molecules. Cl^−^ counterions were added to neutralise the net positive charge of each system. GROMACS version 2019.3 (Van Der Spoel et al., 2005; Pronk et al., 2013) was used for all simulations, using the OPLS-AA forcefield (Jorgensen et al., 1996; Kaminski et al., 2001) for the peptides and the TIP4P model for water molecules (Jorgensen et al., 1983). Fmoc parameters were adapted from Mu et al. (2012). For all simulations, the integration time step was set to 2 fs. Electrostatic interactions were evaluated using the fast smooth particle-mesh Ewald (PME) method with a Coulombic potential cut-off of 1.2 nm (Essmann et al., 1995). For the peptides, covalent bonds involving hydrogen atoms were constrained using the LINCS algorithm (Hess et al., 1997). The systems were firstly energy minimized using the steepest descent algorithm for a maximum of 10,000 steps. Subsequent simulations were performed at 310 K temperature. Each system was equilibrated by running preparatory simulations in which all non-solvent heavy atoms were positionally restrained, firstly under a constant particle number, velocity, and temperature (NVT) ensemble for 100 ps, followed by a constant particle number, pressure, and temperature (NPT) simulation for another 100 ps. In the equilibration simulations, the temperature and pressure were coupled using the Berendsen thermostat and barostat (Berendsen et al., 1984). After the equilibration steps, all restraints were removed, and 800 ns production MD simulations were performed for both WWRR and WPWRR peptide self-assembly systems. For the production simulations, the velocity rescale thermostat of Bussi et al. (2007), with a coupling time constant of 0.1 ps, was used to maintain the temperature at 310 K, while the pressure was maintained at 1 bar using isotropic coupling with the Parrinello-Rahman barostat algorithm (Parrinello and Rahman, 1981). Molecular structures were visualized using Visual Molecular Dynamics version 1.9.3 (VMD) (Humphrey et al., 1996).

### Activity predictions using antimicrobial sequence databases

The probability of antimicrobial activity for the designer tetrapeptide was evaluated using the prediction models Support Vector Machines (SVMs), Random Forests (RF), Discriminant analysis (DA) and Artificial Neural Network classifier (ANN) from the Collection of Anti-Microbial Peptides (CAMP) online (Waghu et al., 2016). The higher the score (max = 1), the greater is the likelihood of the sequence being antimicrobial. Peptides within the score between 0.5 and 1 are considered antimicrobial by those prediction tools. Given that only natural amino acids are considered by the algorithms without any possibility of modification, the Fmoc group was replaced by the aromatic phenylalanine (F) and the C-termini were as free carboxylic acids instead of amidated.

### Minimal inhibitory concentration assays (MIC)

Minimal inhibitory concentration (MIC) assays were performed following a standard broth dilution procedure (Wiegand et al., 2008) in which bacteria were grown in MHB and fungal in SB overnight. The overnight bacterial suspension was adjusted to 10^6^ CFU/ml in broth according to MacFarlane standard. Bacterial solutions were mixed in 96-well plates with the peptide solution to reach final concentrations of peptides ranging from 1,082 to 2 μM, including inhibition control (Indolicidin), growth control, and sterility control. Within each MIC experiment, each peptide was tested in triplicate across this concentration range and repeated on 3 independent days. Media and glassware were sterilized at 120°C for 1 h with an autoclave prior to all bioassays.

### Fluorescence assisted cell sorting (FACS)

FACS assay was performed following the LIVE/DEAD™ BacLight™ Bacterial Viability Kit (L7012) instructions and stablished procedures (Berney et al., 2007; Robertson et al., 2019), in which bacteria were grown in Mueller Hinton Broth and fungal in Sabouraud Broth overnight to a late–log phase. Then, 900 μl of the cell suspension was added to 100 μl of five different concentrations of peptides solution and incubated overnight at 36°C–37°C. On the next day, the suspension was centrifuged at 10,000 × g for 10 min and the supernatant fraction was removed from the samples. The pellets were resuspended with 1 ml of PBS buffer and incubated at room temperature for 1 h, mixing every 15 min. The sample was centrifuged at 10,000 × g for 10 min, the supernatant removed followed by the resuspension of the pellet in 1 ml of PBS. The washing steps were repeated twice. To perform the staining of the cells, equal volumes of SYTO® 9 and propidium iodide were combined in a microfuge tube and 3 µl of the dye mixture was added to each microbial suspension. The sample was incubated at room temperature in the dark for 15 min. The evaluation of cell viability was performed through the calculation of the red to green fluorescence ratio to determine the proportion of live cells in a sample.

### Scanning electron microscopy (SEM) of microbial cells

Pure peptide samples were prepared according to the procedure described in paragraph 2.2. Bacterial samples were prepared by diluting an overnight growth culture of bacteria to OD600 of 0.3–4 and incubating the resultant bacterial solution with the appropriate concentration of peptide for 1 h at 36°C, after which the bacteria were washed four times with PBS by centrifugation, and the resultant bacterial pellets were fixed in a solution of 2.5% glutaraldehyde in PBS for 30 min at 25°C. Control samples were prepared in the same manner but were not incubated with the peptide. The glutaraldehyde solution was washed thrice from the bacteria. The samples were then dehydrated in an incremental manner with 30%–100% EtOH, with a holding time of 15 min for each step, followed by final dehydration in 100% dry EtOH (Hartmann et al., 2010; Glossop et al., 2019). The samples were placed on silicon wafers and coated with iridium to increase its surface conductivity. SEM was performed on a FEI Quanta 200 SEM (2002) operating at 30 kV accelerating voltage.

### Attenuated total reflectance fourier transform infrared spectroscopy (ATR-FTIR)

Data was recorded at 25°C using a Tensor-II spectrometer (Bruker, Germany) equipped with a Bio-ATR attachment (Bruker, Germany) by directly depositing the samples on the ATR crystal as previous reported by our group (Dharmadana et al., 2019). Spectra were collected over 32 scans within 4,000–400 cm^−1^ wavenumber range at a resolution of 4 cm^−1^. Associated water signal was subtracted from the resulting spectra, and secondary derivatives were calculated using OPUS software (Bruker, Germany).

### Polarized light optical microscopy (PLOM)

Polarizing light optical microscopy (PLOM) was performed with a Zeiss Axioskop microscope (Germany) with crossed polarizers at a magnification of × 10 and × 20 on samples conditioned in thin layers between glass coverslip and slide as previously. Images were recorded using a Nikon digital camera and the Zen freeware (bruker, Germany)

### Negative staining for transmission electron microscopy imaging (TEM)

Carbon-coated grids (EMSCF200H-CU-TH, ProSciTech) were glow discharged to render them hydrophilic. A 1–5 μl aliquot of sample was applied to an upturned grid held in anti-capillary forceps, over moist filter paper, and left for 5 min over moistened filter paper to adsorb. Excess liquid was then removed with filter paper, the grid was then inverted onto a drop of 2% PTA stain, pH 6.9 on Parafilm, for 1 min. The grid was removed, the stain wicked away with filter paper and allowed to dry before viewing in the microscope. The samples were examined using Tecnai 12 Transmission Electron Microscope (FEI, Eindhoven, Netherlands) at an operating voltage of 120 kV. Images were recorded using a FEI Eagle 4 k × 4 k CCD camera and AnalySIS v3.2 camera control software (Olympus).

### Cryogenic transmission electron microscopy (cryo-TEM)

A laboratory-built humidity-controlled vitrification system was used to prepare the samples for Cryo-TEM. Humidity was kept at 80% for all experiments, and ambient temperature was 22°C. 300-mesh copper grids coated with perforated carbon film (Lacey carbon film: ProSciTech GSCU300FL-50, Kirwan, Qld, Australia) were glow discharged using a Pelco EasiGlow Glow Discharge unit to render the carbon film hydrophilic. 3 μl aliquots of the sample were pipetted onto each grid prior to plunging. After 5 s adsorption time the grid was blotted manually using Whatman 541 filter paper, for 4 s. The grid was then plunged into liquid ethane cooled by liquid nitrogen. Frozen grids were stored in liquid nitrogen until required. The samples were examined using a Gatan 626 cryoholder (Gatan, Pleasanton, CA, USA) and Tecnai 12 Transmission Electron Microscope (FEI, Eindhoven, Netherlands) at an operating voltage of 120 kV. At all times low dose procedures were followed, using an electron dose of less than 5 electrons/Å^2^ for all imaging. Images were recorded using a FEI Eagle 4 k × 4 k CCD camera at a range of magnifications using AnalySIS v3.2 camera control software (Olympus).

### Cryogenic scanning electron microscopy (cryo-SEM)

Carbon-coated Lacy Formvar 300-mesh copper grids (GSCU300FL-50, ProSciTech, Australia) were glow discharged to render them hydrophilic. A 1 μl drop of peptide sample (5% w/w) at day 19 was applied on an upturned grid held in place by a tweezer. This was then turned upside down and gently smeared the sample on a Parafilm ‘M’. The samples were vitrified by plunging them into liquid ethane, which was pre-cooled in liquid nitrogen. The vitrified samples were stored within a precooled grid holder. The grids were transferred onto the brass grid holder under liquid nitrogen. The grid holder with samples was transferred in liquid nitrogen slush under vacuum into the cryogenic preparation chamber (Gatan ALTO 2500, UK) using the transfer arm. In the preparation chamber, the samples were sublimed at −80°C for 60 min and subsequently coated using platinum at ≤ −140°C for 120 s using a current of 10 mA. The cryogenically processed samples were transferred under vacuum onto the liquid nitrogen cooled stage within the Field Emission Scanning Electron Microscope (FE-SEM) chamber for imaging. The samples were imaged using a Zeiss Sigma 300 VP SEM (Germany) operated in the secondary electron (SE) mode. An accelerating voltage of 2 kV with a probe current of 80 pA was used for imaging. The location for imaging was taken in the region of 200 nm from the edge of the grid beyond the grid holder.

### Synchrotron small-angle and wide-angle X-ray scattering (SAXS/WAXS)

Samples were prepared within wax-sealed quartz glass capillaries of 0.01 mm wall thickness and 1.5 mm outer diameter (Hilgenberg GmbH, Germany). SAXS and WAXS experiments were performed on the SAXS/WAXS beamline operated at 11 KeV at the Australian Synchrotron (Melbourne, Australia). The capillaries were introduced into a capillary holder accommodating 20 capillaries and the temperature was controlled at 25°C and at 37°C. The SAXS/WAXS patterns were recorded for a sample-detector distance of 1.6 m with an exposure time of 1 s, providing a q range of 0.01–1.5 Å^−1^. Scattering patterns were recorded using 1 M Pilatus detector for SAXS and a 200 k Pilatus detector for WAXS. Each pattern resulted from radial integration and subtraction of solvent scattering using the in-house Scatterbrain (Synchrotron). The SaSView (SaSView application) application and Primus suite (Konarev et al., 2003) were used to perform the Guinier and form factor analysis. The hexagonal parameter (a) was calculated from the position q) of the first diffraction peak using Eq. 1 (Alexandridis et al., 1998).

$$Q=4\pi /\left(a\sqrt{3}\right)$$ (1)

### Rheology

Rheology was undertaken on a calibrated rheometer (Anton Paar MCR 301, Anton Paar Pty. Ltd. Austria) fitted with a 15 mm cone plate fixture with a cone angle of 1° (CP15-1, Anton Paar Pty. Ltd. Austria). Shear strain tests were collected between 0.05% and 100% with a constant angular frequency of 3 rad·s^−1^. All measurements were carried out in triplicate with the mean and standard deviation shown, calculated and plotted in GraphPad Prism.

## Results

### Rational design of bi-functional peptide sequences

**FIGURE 1:**
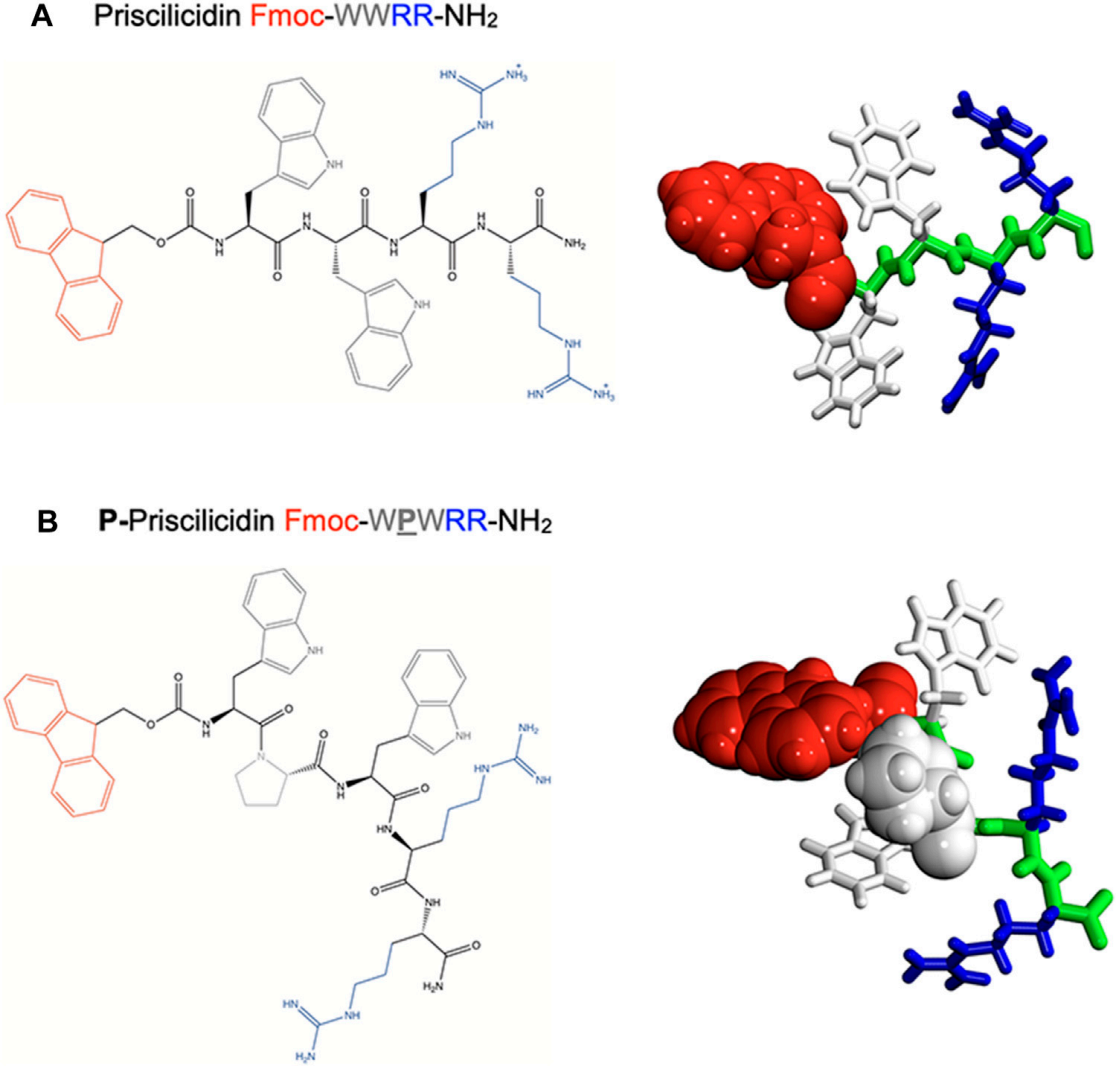
Molecular structure of the rationally designed peptides **(A)** Priscilicidin and **(B)** P-Priscilicidin. Left panel: developed amino-acid sequences. Right panels: *ab-initio* monomers for molecular dynamics simulations.

To design a simple chemical sequence that minimises the cost of synthesis, we chose a combination of natural aromatic and cationic residues to target the microbial lipid membrane, through respectively: i) hydrophobic insertion within the lipid bilayer, ii) electrostatic interactions with membrane phospholipid head groups and other microbial anionic compounds, such as lipopolysaccharides or teichoic acid respectively found in Gram-negative and Gram-positive bacteria cell wall. The high occurrence of two repeats of tryptophan (W, aromatic) and arginine (R, cationic) residues in natural AMPs led us to consider double repeats of each of these amino-acids (Cardoso et al., 2021). A typical example is the potent natural antimicrobial peptide indolicidin, isolated from bovine neutrophils and of sequence ILPWKWPWWPWRR-NH_2_, which contains seven residues as tryptophan or arginine over a total of thirteen amino-acids (Selsted et al., 1992; Subbalakshmi and Sitaram, 1998; Batista Araujo et al., 2022). The antimicrobial activity of the tryptophan-arginine motif in natural AMPs is generally rationalised by the arginine guanidinium group that can either interact with negatively charged microbial membranes *via* electrostatic interactions or participate in cation–π interactions with the indole group of tryptophan. This duality is believed to favour AMP penetration into the microbial lipid bilayer (Chan et al., 2006; Saravanan et al., 2014). In our designer sequences, we hence included W and R repeats (Figure 1).

Capping the charged peptide termini by neutral and aromatic groups was included in our sequences to optimize the polarity and amphiphilic properties of the peptide, as critical physicochemical features for self-assembly and antimicrobial propensity. In fact, these modifications enhance the segregation of aromatic groups (Fmoc, W) from charged-aliphatic groups (R, amide) in the peptide primary sequence. Common chemical modifications of peptide termini were chosen, respectively amidation found in natural sequences, e.g., indolicidin, and the synthetic fluorenylmethoxycarbonyl (Fmoc) group used as a common conjugation step in peptide solid phase synthesis. Amidation was further reported to limit enzymatic proteolysis and enhance stability (Sforça et al., 2004; Mura et al., 2016). The insertion of the highly aromatic Fmoc at the N-terminal was expected to also favour peptide self-assembly through π-stacking, as previously reported for single to tetrapeptide Fmoc-containing sequences (Smith et al., 2008; Orbach et al., 2009), including antimicrobial sequences based on the Fmoc-FF motif or bearing unnatural amino-acids (McCloskey et al., 2017; Schnaider et al., 2017; Misra et al., 2021; Prakash et al., 2022).

In accordance with reported AMP motifs, the resulting designer tetrapeptide Fmoc-WWRR-NH_2_ (Figure 1A) exhibits a net charge of +2 for a wide range of pH, a rich content in aromatic and cationic residues as well as a highly polar and amphiphilic sequence. To test the influence of backbone conformation in our approach, we designed a second peptide, P-Priscilicidin (Figure 1B) that includes a proline turn to disrupt backbone linearity and potentially peptide self-assembly. The amino-acid sequence of P-Priscilicidin corresponds to the C-terminal sequence of indolicidin, a natural potent antimicrobial peptide biosynthesised by bovine neutrophils (Selsted et al., 1992; Subbalakshmi and Sitaram, 1998; Batista Araujo et al., 2022).

### Predicted *versus* experimental antimicrobial activity

The designer Fmoc-peptides were submitted to online antimicrobial prediction algorithms embedded within the CAMP database of antimicrobial peptides. A phenylalanine was used *in lieu* of Fmoc as these tools only consider natural amino-acids (Waghu et al., 2016). All prediction algorithms returned a good probability of antimicrobial activity for both peptides when compared to the indolicidin control; except for the RF algorithm that returned a less probable activity (Table 1). Given the approximation in sequences and the high dependence on the algorithm data source, this difference cannot be easily explained.

**TABLE 1:** Antimicrobial activity predictions from different algorithms in the CAMP database.

Peptide sequence in database	SVM	RF	ANN	DA
**Indolicidin-like** ILPWKWPWWPWRR (free N, C-termini)	0.999	0.991	AMP	0.997
**Priscilicidin (Pris) -like** (F)-WWRR (free N, C-termini)	1.000	0.568	AMP	0.997
**P-Priscilicidin (P-Pris) -like** (F)-WPWRR (free N, C-termini)	1.000	0.572	AMP	0.972

Green data indicate prediction for antimicrobial activity. Red data indicate prediction for non-antimicrobial activity.

The antimicrobial activities of the Priscilicidin, P-Priscilicidin were experimentally tested on bacterial and fungal strains using Minimum Inhibitory Concentration (MIC) assays and verified by Fluorescence Assisted Cell Sorting (FACS) assays for the most critical strains, including the resistant ones (Table 2; Supplementary Table S1). Indolicidin was used as a control. The FACS results are here given as the minimum concentration showing over 90% dead microbes, while the full results are provided in Supplementary Table S1; Supplementary Figures S2–S16.

**TABLE 2:** Experimental antimicrobial activities of indolicidin (control), Priscilicidin (Fmoc-WWRR-NH2) and P-Priscilicidin (Fmoc-WPWRR-NH2) from minimum inhibitory concentration assays (MIC) and verified by fluorescence-assisted cell sorting assays (FACS) for the most critical strains (results given for >90% dead cells).

		Gram-negative bacteria		Gram-positive bacteria				Fungi	
Peptide sequence	Assay	E. *coli*	*P. aeruginosa*	*E*. *faecalis*	*E*. *faecium*	*S*. *aureus/Methicillin resistant*	*S. pneumoniae*	*C*. *albicans*	*C*. *auris* Fluconazole resistant
**Indolicidin (control)** ILPWKWPWWPWRR-NH_2_	MIC FACS	16 μM 10 μM	33 μM	33 μM	33 μM	16/33 μM >42 μM I	16 μM	4 μM	16 μM 21 μM
**Indolicidin C-terminus** WPWRR-NH_2_	MIC	313 μM	313 μM	*N/A*	*N/A*	*N/A*	*N/A*	*78 μM*	*313 μM*
**Di-Tryptophan** **(control)** WW	MIC	*N/A*	*N/A*	*N/A*	*N/A*	*N/A*	*N/A*	*N/A*	*N/A*
**Priscilicidin (Pris)** Fmoc-WWRR-NH_2_	MIC FACS	68 μM 54 μM	68 μM	68 μM	**34 μM**	**17/34 μM 27 I (R)**	**17 μM**	**8 μM**	**17 μM 24 μM**
**P-Priscilicidin (P-Pris)** Fmoc-WPWRR-NH_2_	MIC FACS	122 μM 196 μM	61 μM	61 μM	61 μM	**31**/61 μM 196 μM R)	**31 μM**	**15 μM**	**31 μM** 54 μM

Bold values indicate potent antimicrobial activity when compared to the indolicidin control. Italic data indicate low antimicrobial activity when compared to indolicidin control. N/A stands for non-applicable and corresponds to values >1,000 μM.

Priscilicidin exhibited antimicrobial activity in the low micromolar range (under 50 μM) against Gram-positive bacteria (*E*. *faecium*, *S*. *aureus*, methicillin-resistant-*S. aureus*, *S*. *pneumoniae*) and fungal strains (*C*. *albicans* and fluconazole-resistant *C*. *auris*), with similar inhibitory concentrations to the indolicidin control. However, the Priscilicidin peptide was found less active against representative Gram-negative bacteria (*E*. *coli*, *P*. *aeruginosa*) with MICs and FACS results in the high micromolar range. Similar values were obtained for P-Priscilicidin but for *E*. *faecium* and resistant strains.

**FIGURE 2:**
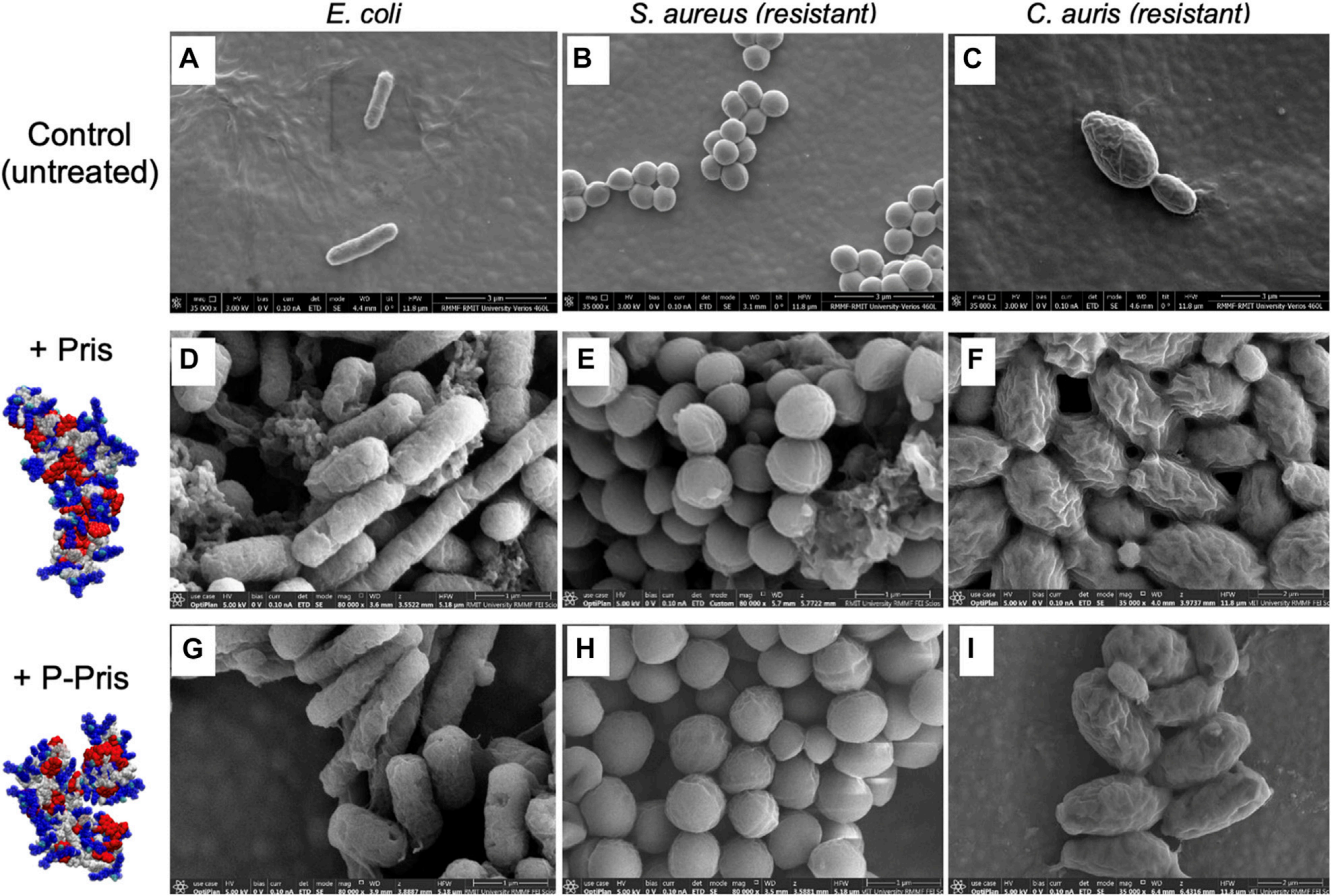
Representative scanning electron micrographs (SEM) of untreated (control, **A–C**) and treated bacterial/fungal cells with Priscilicidin **(D–F)** and P-Priscilicidin **(G–I)**. Priscilicidin was added at 2.4 times MIC **(D–F)** and the peptide P-Priscilicidin at 1.2 times MIC **(G–I)** (other concentrations are shown in Supplementary Figures S17–S25).

For one representative strain of each microbial type, activity assays were complemented with direct observations of the peptide impact on microbial cells using scanning electron microscopy. A range of peptide concentrations was tested, including the inhibitory concentration found by MIC and FACS assays for each strain (Figure 2; Supplementary Table S1; Supplementary Figures S17–S25). For peptide concentrations above the inhibitory concentrations obtained by MIC and FACS assays, electron micrographs revealed the cells of E. *coli* (Gram-negative bacteria), S. *aureus* (Gram-positive bacteria) and fluconazole-resistant C. *auris* (fungi) to exhibit i) higher propensity to agglomerate and ii) damaged or perforated cell membranes, as evidenced by the numerous observed defects at the cell surfaces compared to control samples. These observations hence reveal the peptides to impact the structure of the microbial cell membranes.

Altogether, activity assays (MIC, FACS) and electron microscopy of treated microbes reveal that the rationally designed peptides Priscilicidin and P-Priscilicidin display a similar antimicrobial activity to the potent indolicidin peptide for representative Gram-positive bacteria and fungi, besides significantly shorter sequences. Interestingly, the addition of the Fmoc-moiety at the C-terminus significantly enhanced antimicrobial activity, as shown by the lower activity of the indolicidin C-terminus sequence when compared to P-Priscilicidin (Table 2). The linearity of the Priscilicidin backbone further provides increased activity (Table 2; Figure 2). To summarise, the antimicrobial activity of the short sequences can be ranked as: WPWRR-NH_2_ (indolicidin C-terminus) < Fmoc-WPWRR-NH_2_ (P-Pris) < Fmoc-WWRR-NH_2_ (Pris).

### Molecular dynamics support sequence-controlled peptide self-assembly

**FIGURE 3:**
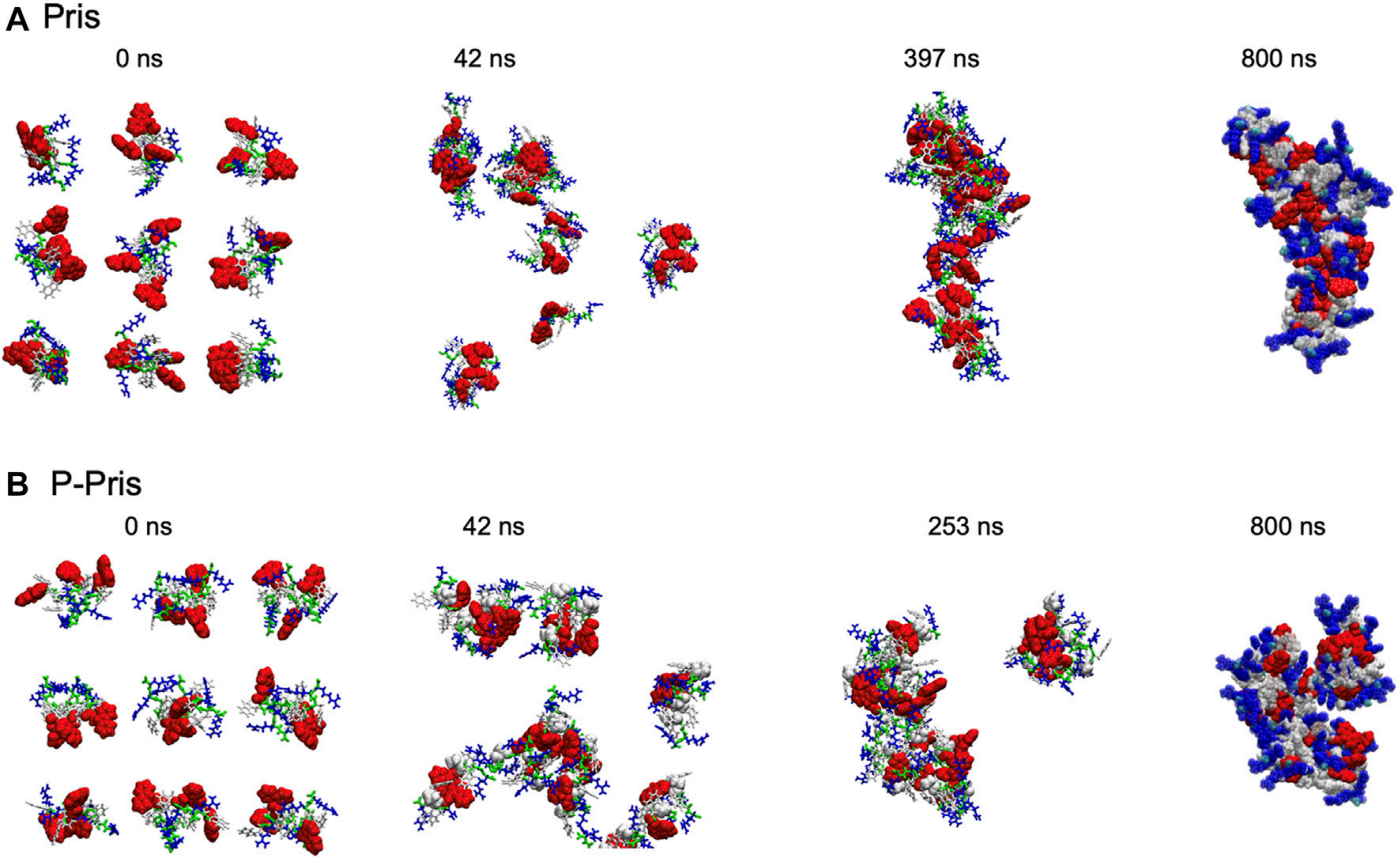
Priscilicidin **(A)** and P-Priscilicidin **(B)** molecular dynamics simulations.

**FIGURE 4:**
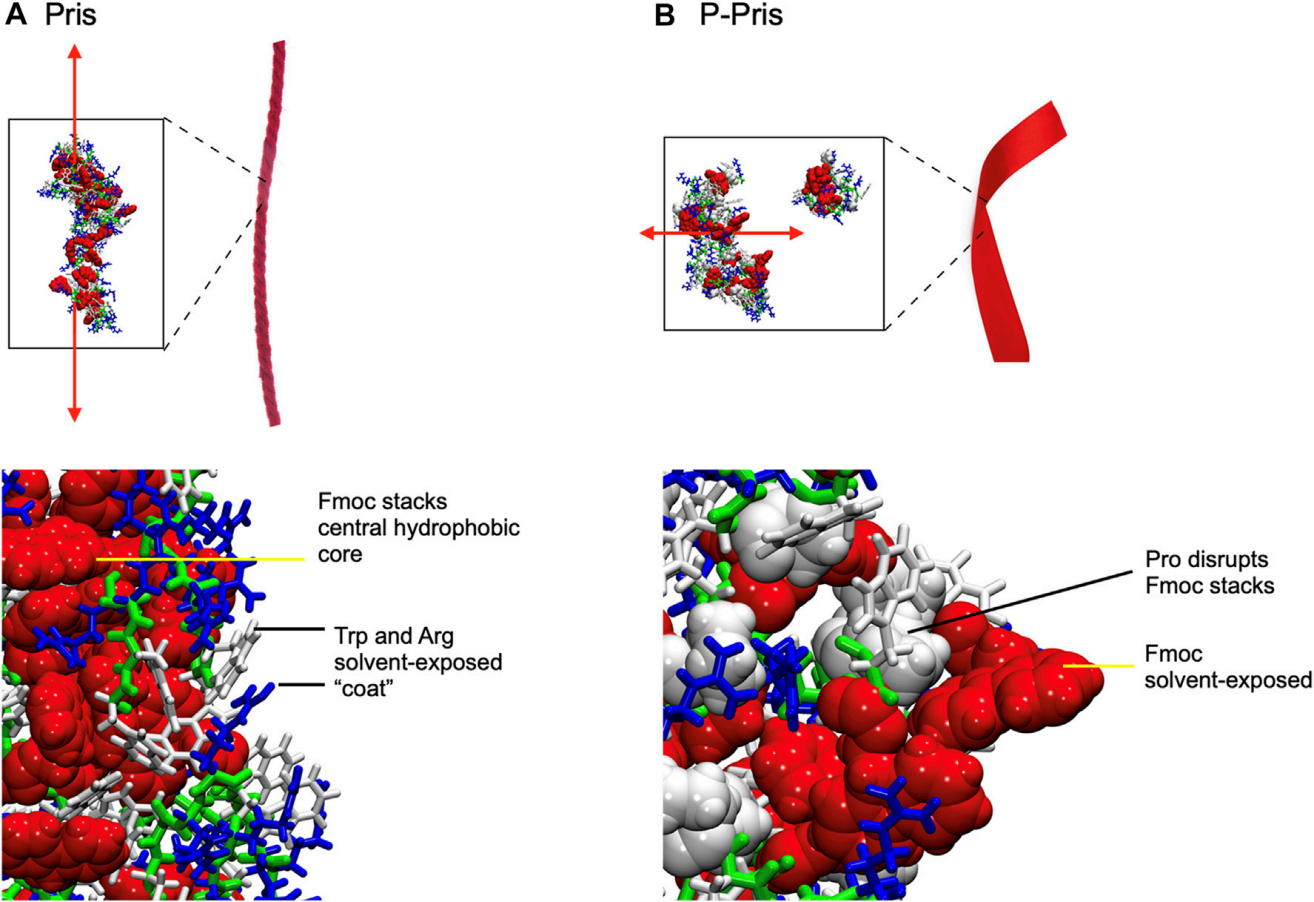
Final Molecular Dynamics simulation snapshots of Priscilicidin **(A)** and P-Priscilicidin **(B)**, with conceptual cartoon representations of extended fibrils (red ribbons) drawn for illustration only. Fmoc groups form partially ordered ring stacks in Priscilicidin, sequestered from solvent by Trp and Arg **(A)**, which in contrast are disordered and solvent-exposed in P-Priscilicidin **(B)**.

Molecular dynamics simulations were performed on the designer peptides to verify the self-assembly principles hypothesized in our rational sequence design (Figures 3, 4).

Molecular dynamics simulations up to 397 ns predicted the self-assembly of Priscilicidin into extended chains, stabilised by continuous intermolecular pi-stacking of the aromatic and hydrophobic Fmoc groups in the central core of the structure (Figures 3A, 4A). The Pris extended oligomers displayed a Tryptophan and Arginine coat protecting the Fmoc hydrophobic core structure. Further simulation to 800 ns further stabilised the elongated Fmoc stacks and displayed limited lateral growth of the oligomers, supporting elongation into nanofibrils.

For P-Priscilicidin, molecular dynamics predicted the proline turn to disrupt the formation of continuous Fmoc stacks, leading to partial clumping of oligomers with discontinuous intermolecular Fmoc pi-stacking (Figures 3B, 4B). The resulting oligomeric structure displayed Fmoc groups that are partially exposed to the solvent and not exclusively located within a hydrophobic core as for Priscilicidin. These simulations support the formation of P-Pris globular oligomeric species with unfavored elongation into nanofibrils or nanostructures.

### Molecular spectroscopy insights into arginine side-chain solvent exposure

The peptide conformation in solution was experimentally characterized by attenuated total reflectance Fourier-transform infrared spectroscopy (ATR-FTIR), as a function of peptide acetate concentration in water and time after sample mixing at room temperature (Table 3; Supplementary Figure S26). For ATR-FTIR, analysis was focused on the amide I and Arginine range of vibrations (Venyaminov and Kalnin, 1990; Surewicz et al., 1993). The main difference between IR spectra was observed on the arginine side-chain vibrations, being recorded as either i) two vibrations with a smaller split between wavenumber values (around 1,680 + 1,630 cm^−1^), similarly to reported vibrations for single amino-acid arginine side-chain in solution (around 1,670 + 1,630 cm^−1^); which were assigned to solvent-exposed arginine side-chain in peptide monomer or globular oligomer, as indicated by molecular dynamics; or ii) two vibrations with a larger split between wavenumber values, similarly to arginine side-chain buried within protein hydrophobic pockets; which were interpreted as arginine side-chain buried within the core of peptide Fmoc-stacked assemblies, as indicated by molecular dynamics (Table 3; Supplementary Figure S26) (Venyaminov and Kalnin, 1990; Surewicz et al., 1993).

**TABLE 3:** ATR-FTIR shifting vibrations in the amide I region (1,700–1,600 cm^−1^), assigned as solvent-exposed arginine side-chain within peptide monomer/small oligomer (blue) or arginine side-chain buried within peptide self-assembly Fmoc-stacked core (orange).

			Single amino-acid arg vibrations (cm^−1^)	Protein arg vibrations (cm^−1^)	
		References	1,672–1,673 + 1,633–1,636	1,688–1,695 + 1,576–1,577	
Peptide sequences	Conc (% wt)	Time after mixing	Solvent-exposed Arg	Buried Arg within hydrophobic pocket	Interpretation for peptide assembly according to MD
**Priscilicidin (Pris)** Fmoc-WWRR-NH_2_	1	0	1,680 + 1,634		monomer/globular oligomer
		3 days	1,680 + 1,630		
		5 days	1,684 + 1,632		
		30 days	1,680 + 1,635		
	5	0	1,680 + 1,629		monomer/globular oligomer
		3 days		1,694 + 1,634	Fmoc-stacked assembly
		5 days		1,695 + 1,634	
		30 days		1,695 + 1,634	
	10	0	1,679 + 1,629		monomer/globular oligomer
		3 days		1,694 + 1,634	Fmoc-stacked assembly
		5 days		1,695 + 1,634	
		30 days		1,695 + 1,634	
**P-Priscilicidin (P-Pris)** Fmoc-WPWRR-NH_2_	1	0	1,670 + 1,629		monomer/globular oligomer
		30 days	1,680 + 1,634		
	5	0	1,679 + 1,629		
		30 days	1,679 + 1,630		
	10	0	1,678 + 1,629		
		30 days	1,679 + 1,630		

For Priscilicidin, the IR data support the formation of peptide self-assemblies with Arginine side-chains embedded within a hydrophobic core–i.e., Fmoc-stacks according to molecular dynamics–(MD)—with concentration and time increase. This is consistent with a cooperative intrinsic self-assembly process based on Fmoc-stacking as indicated by molecular dynamics. However, IR data indicate that P-Priscilicidin data does not undergo such a process but remains in a state with solvent-exposed Arginine sidechains, consistently with molecular dynamics.

### Experimental evidence of molecular self-assembly into oligomers, nanoribbons and nanofibrils

**FIGURE 5:**
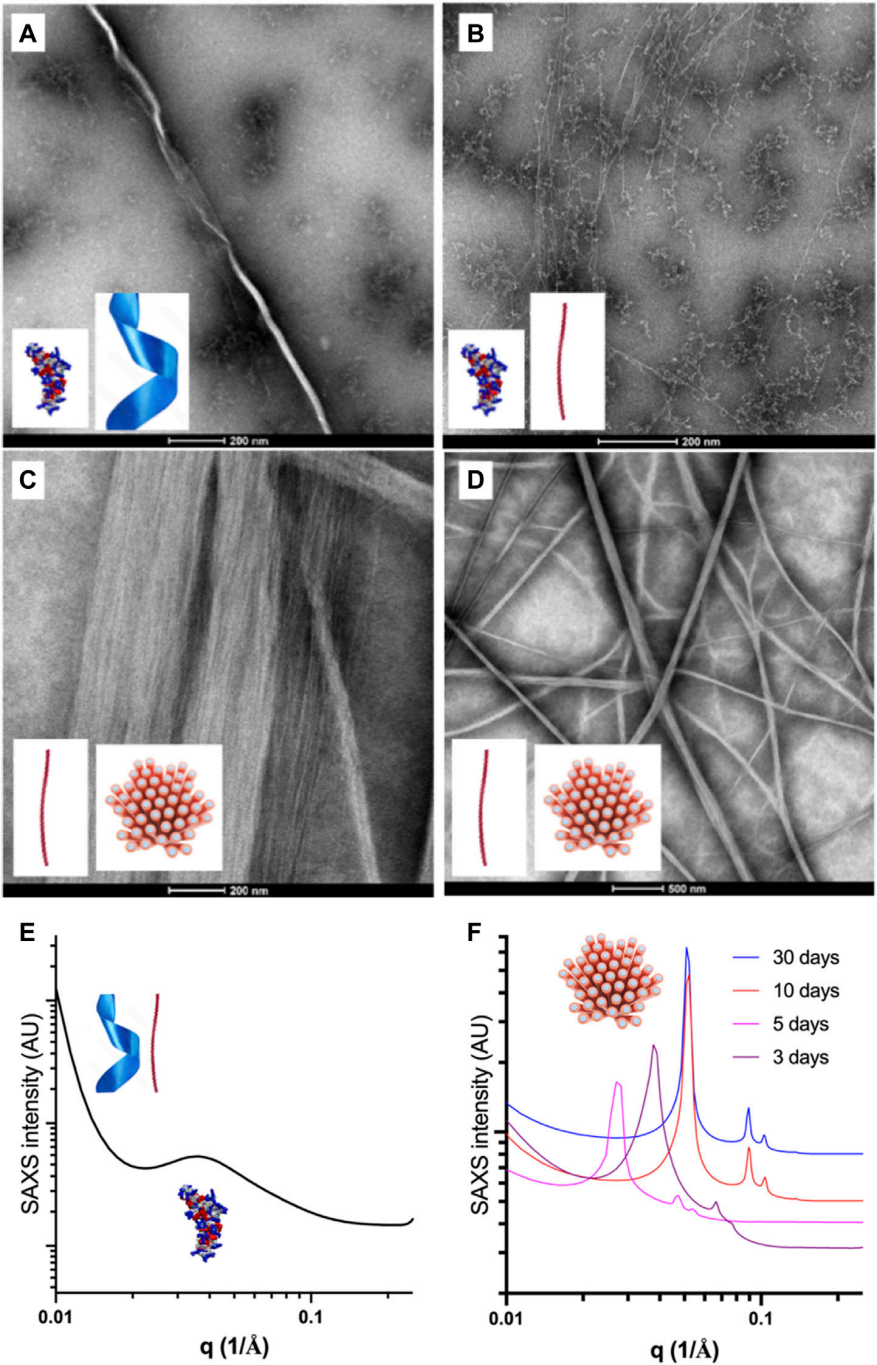
Representative transmission electron micrographs and SAXS patterns of the self-assembled species formed in water by Priscilicidin (Fmoc-WWRR-NH_2_) over the concentration-time space. **(A)** Nanoribbon and oligomers for 1% wt Pris ac, 1 week. Scale bar 200 nm; **(B)** Nanofibrils and extended oligomers for 5% wt Pris ac < 12 h. Scale bar 200 nm; **(C)** Single and aligned nanofibrils (into nanofibers) for 5% Pris ac, 1 week. Scale bar 200 nm; **(D)** Single and aligned nanofibrils (into nanofibers) for 5% Pris ac, 7 days. Scale bar 500 nm. **(E)** SAXS patterns recorded at 25°C for 1% Pris ac, 30 days interpreted as a coexistence of oligomers, nanoribbons and nanofibrils (Table 4); **(F)** SAXS patterns recorded at 25°C for 10% Pris ac as a function of time interpreted as maturing hexagonal networks of nanofibrils (Table 4).

**FIGURE 6:**
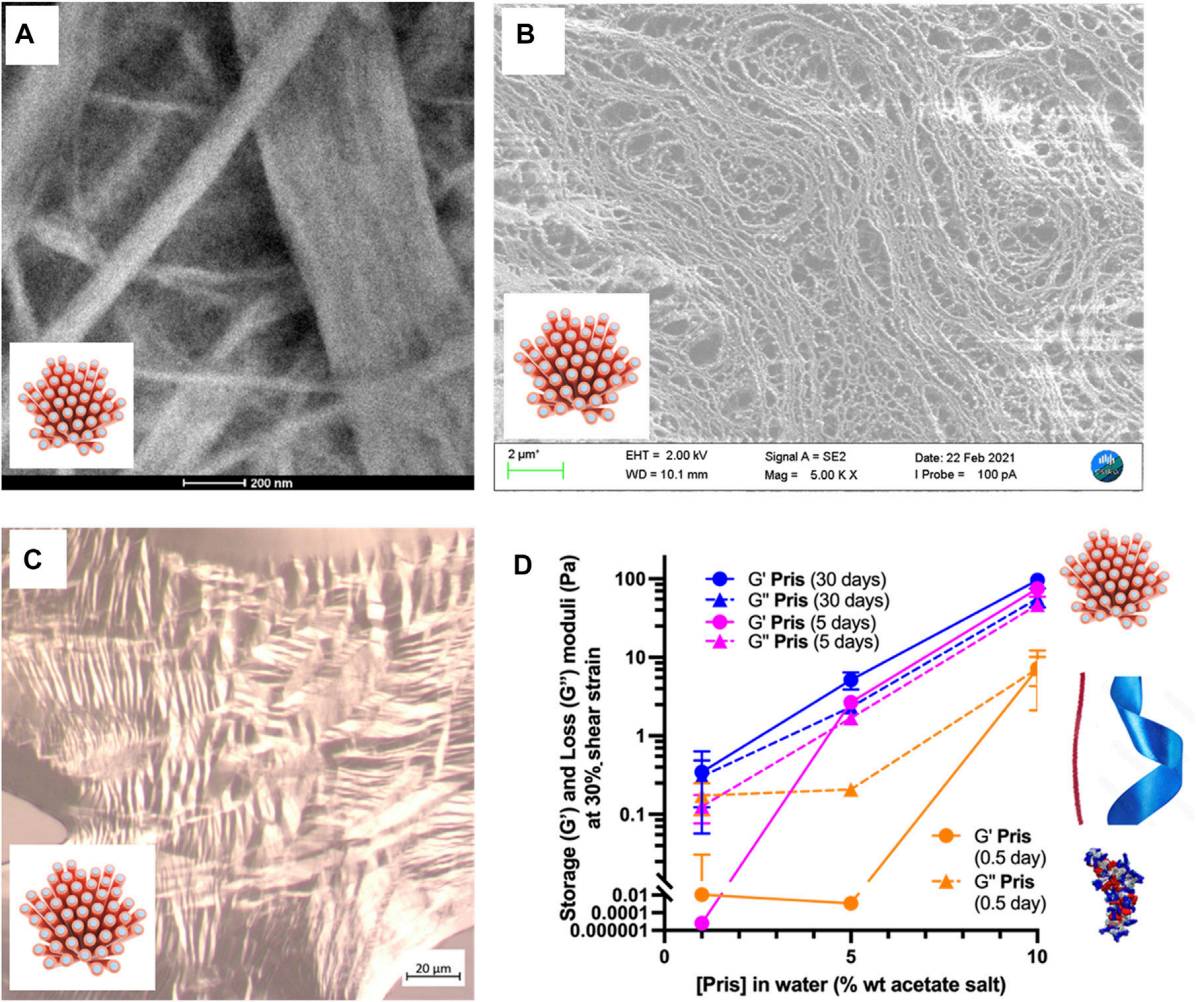
Priscilicidin nanostructured samples: mesophasic and viscoelastic properties. **(A)** 10% at t = 5 days. Scale bar 200 nm; **(B)** 5% at t = 5 days. Scale bar 2 μm; **(C)** 10% w/w hydrogel (3 days old), dried on slide showing nematic optical textures. Scale bar 20 μm; **(D)** G’ and G” values at 30% strain over Priscilicidin concentration and time space.

Transmission electron microscopy imaging of the peptide samples in water was performed for [1–10] % wt peptide acetate from a few hours to 1 month of incubation at 4°C–5°C, using the negative staining technique (TEM) and cryogenic sample preparation (cryoTEM, cryoSEM) (Figures 5, 6, Supplementary Figure S27).

For short incubation times of a few hours, three different Priscilicidin oligomeric structures were imaged by transmission electron microscopy as a function of peptide concentration (Figure 5; Supplementary Figure S26): i) extended oligomers, as predicted by molecular dynamics (background in Figures 5A, B), ii) nanoribbons and iii) nanofibrils, consistently with bidirectional assembly of the extended oligomers (negative staining in Figures 5C, D, cryo-TEM in Supplementary Figure S27). The critical concentration of self-assembly is below 1% wt, while for the highest concentrations (5%, 10% wt), the nanofibrils were observed to associate into nanofibers and large bundles by tight close contacts (Figures 5C, D, 6A,6B). These observations support the nanosheets to be transient species towards the formation of nanofibrils and nanofibers. We hence propose Priscilicidin self-assembly to proceed from extended oligomers to nanosheets, which fold into nanofibrils within a few hours. These nanofibrils then align into large clusters that can tighten into nanofibers.

Synchrotron small angle X-ray scattering was recorded at room temperature (25°C) and at 37°C for [1–10] % wt Priscilicidin and P-Priscilicidin (as acetate salts) in water, for incubation times of a few days to 1 month, as kept at 5°C in between experiments (Figures 5E,F; Supplementary Figures S28–S29; Table 4).

**TABLE 4:** Small angle X-ray scattering fits and assignments for Priscilicidin as a function of peptide acetate concentration in water, temperature, and incubation time.

[Pris ac]% wt in water	Incubation time at 5°C	Structure factor	SAXS slope q^−*x*^ x)	Lattice parameter (a_hex_) nm	Interpretation on peptide assemblies (Dharmadana et al., 2020)
1	3 days	None	1.57 (25°C)|1.77 (37°C)	N/A	Coexistence of q^−1^: rod-like oligomers/nanofibrils and q^−2^: flat 2D-structures/nanoribbons
	10 days		1.44 (25°C)|1.51 (37°C)		
	30 days		1.56 (25°C)|1.69 (37°C)		
5	3 days	Hexagonal network	N/A	15 (25°C)|14 (37°C)	Maturing hexagonal networks of laterally aligned nanofibrils (nanofibers)
	10 days			15 (25°C)|14 (37°C)	
	30 days			15 (25°C)|15 (37°C)	
10	3 days	Hexagonal network	N/A	13 (25°C)|13 (37°C)	Maturing hexagonal networks of laterally aligned nanofibrils (nanofibers)
	10 days			14 (25°C)|13 (37°C)	
	30 days			14 (25°C)|13 (37°C)	

Consistently with the molecular dynamics, molecular spectroscopy and TEM, SAXS showed Priscilicidin to undergo self-assembly into extended oligomers, nanoribbons, nanofibrils and hexagonally-aligned nanofibrils into nanofibers, with concentration and time increase.

**FIGURE 7:**
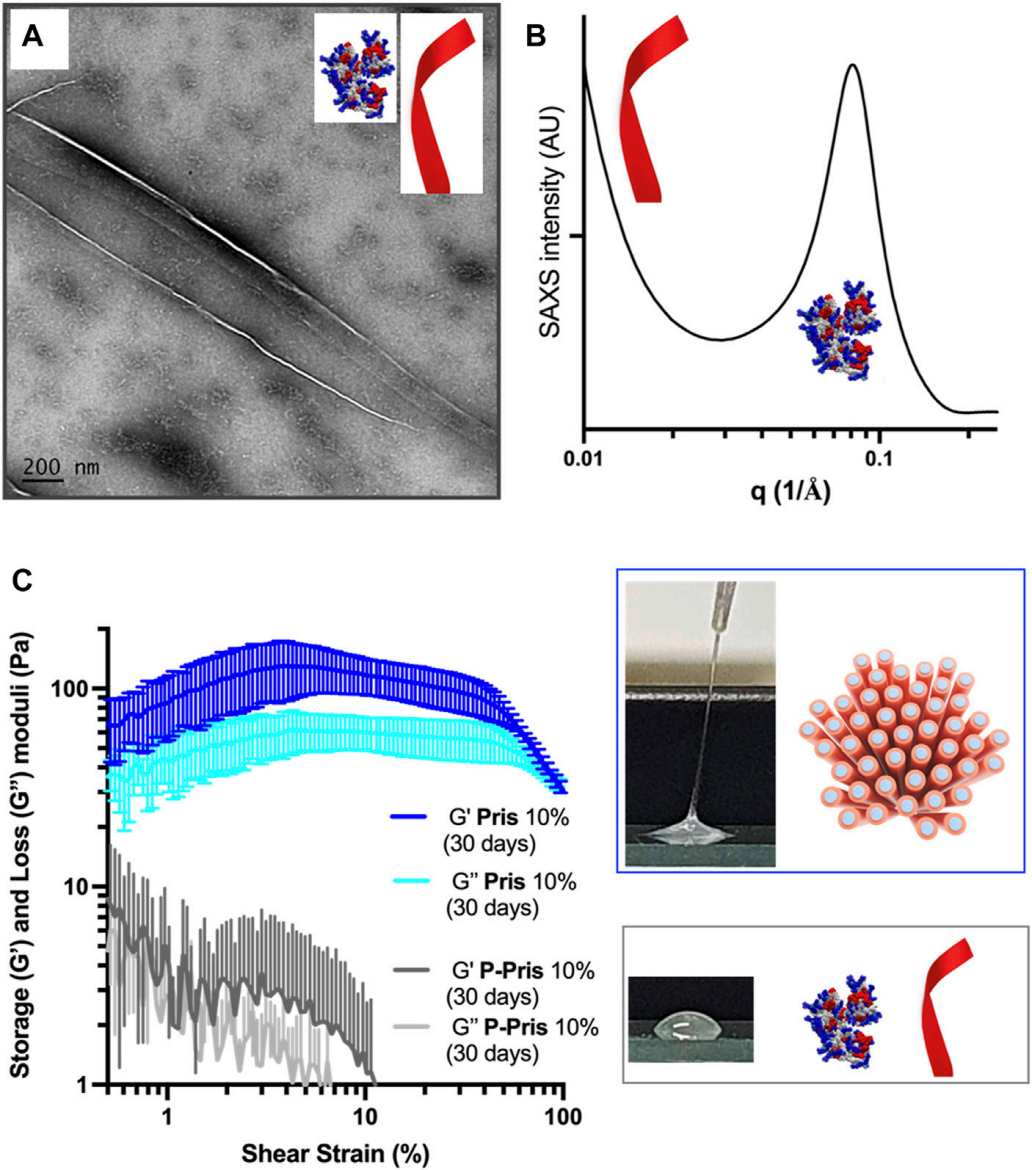
Peptide species **(A,B)** and rheological behaviour **(C)** of 10% wt P-Priscilicidin pure water solutions aged for 30 days at 5°C compared to **(C)** the rheological behaviour and macroscopic appearance of 10% wt Priscilicidin hydrogels aged under the same conditions. Image **(A)**: scale bar 200 nm.

For P-Priscilicidin (Figure 7A; Supplementary Figure S30–S31), the only nanostructures that could be imaged were nanoribbons for the highest concentration tested (10% wt in water) after 1 month incubation, in coexistence with globular oligomers. Consistently, SAXS patterns did not show any form or structure factor, but a correlation peak and a SAXS slope supporting the formation of oligomers and nanoribbons for 10% wt peptide acetate in water after 1 month (Figure 7B).

### Sequence-controlled hydrogelation into nanostructured mesophases

Priscilicidin hydrogel characterisation was performed using electron microscopy, polarised light optical microscopy, and oscillatory rheology (Figure 6; Supplementary Figure S32). For incubation times of a few days, Priscilicidin was observed to spontaneously form homogeneous hydrogel, from around 5% w/w of peptide acetate in pure water. The peptide could be easily solubilised in pure water at concentrations of at least 10% w/w (higher concentrations not explored). The sample then developed over time viscoelastic and self-healing gel behaviour when manipulated (Figure 6C). After 1 month of incubation, transmission electron microscopy (TEM, Figure 6A) on dried 10% Priscilicidin samples confirmed the formation of a few microns long, semi-flexible nanofibers resulting from nanofibrils in close contact. The formation of nanofibril bundles is consistent with the hexagonal networks revealed by SAXS, while the nanostructure semi-flexibility supports liquid crystalline properties. The mesophasic state of Priscilicidin hydrogels was further supported by the observation of birefringence and nematic optical textures under polarised light microscopy (Figure 6C). Cryogenic scanning electron microscopy (cryoSEM, Figure 6B) revealed reticulation of the whole hydrogel volume by nanofibrillar structures, hence confirming the hydrogel homogeneity.

The rheological behaviour of Priscilicidin (Figures 6D, 7C) and P-Priscilicidin (Figure 7C) was characterised through measuring the storage (G′) and loss (G″) moduli as a function of peptide concentration and time. The peptides generally had a non-linear behaviour with time, assigned to unfinished self-assembly process from monomers to oligomers to nanoribbons (both peptides), to nanofibrils or mature nanofibers (Priscilicidin) as evidenced above by electron microscopy and SAXS. The Priscilicidin samples rapidly assemble and continue to form a soft gel network from day 0 to day 5. When comparing the rheological data from day 5 to day 30, there is no looking at the significant increase in gel formation after day 5 except for the 1% wt Priscilicidin sample (Figure 6D; Supplementary Figure S29). On the contrary, P-Priscilicidin forms very weak and unstable gels under shear strain (Figure 7C; Supplementary Figure S29).

In summary, Priscilicidin forms viscoelastic nanostructured mesophases, while P-Priscilicidin forms colloidal solutions of low viscosity.

## Discussion and conclusion

Molecular dynamics simulations predicted Priscilicidin to assemble in water into small oligomers and nanofibrils, through a balance of aromatic stacking, amphiphilicity and electrostatic repulsion. The resulting extended oligomer consists of solvent protected Fmoc at the core, protected by an exposed hydrophilic layer of Arg and Trp. It is noteworthy that a similar supramolecular assembly of ordered, elongated Fmoc stacking was previously observed in simulations of a prototypical dipeptide, Fmoc-AA (Mu et al., 2012; Guo et al., 2015). ‘Worm-like’ extended nanostructures have also been observed to form in water for Fmoc-FF and Fmoc-FFD (Li et al., 2018). The appearance of a broadly similar superstructure in Priscilicidin (Fmoc-WWRR-NH_2_), with an amino acid sequence distinct from these previously studied peptides, highlights the vital contribution of the Fmoc-Fmoc interaction in peptide self-assembly. On the other hand, we have demonstrated that introduction of a single proline, in the form of P-Priscilicidin, is sufficient to alter the structures of Pris monomers, ultimately translating into formation of a far less ordered, roughly globular oligomeric complex. This result highlights the importance of structurally disruptive residues on reducing the fibrillogenic potential of peptides which otherwise strongly favour elongation.

Fmoc-FF–a reference ultrashort self-assembling Fmoc-peptide–is a poorly water-soluble substance. Complete solubilisation for homogeneous lyotropic self-assembly of Fmoc-FF in water can only be achieved by i) dissolution in dimethyl sulfoxide (DMSO) followed by a dilution step in water and change of pH, or ii) by heating the peptide initial aqueous solution up to 90°C for peptide solubilisation before a cooling step (Smith et al., 2008; Tang et al., 2009). Depending on conditions, Fmoc-FF displays polymorphism between a hydrogel state and precipitates (Tang et al., 2009). Comparatively, the Fmoc peptides reported here display a largely improved water solubility and sample physicochemical stability. From a pharmaceutical sciences point of view, these new peptides offer the advantage of a simple hydrogel preparation protocol, under standard soft mixing conditions, with no observed polymorphism nor precipitation, for at least 1 month after preparation.

We here provide a proof of concept for bi-functional molecular design on Fmoc-peptides containing 4 and 5 amino-acids. Using minimalistic molecular engineering principles, we designed peptides that form antimicrobial hydrogels of different viscoelastic properties. Given the broad applications of peptide nanostructured hydrogels as, for instance, pharmaceutical or cosmetic biomaterials, our results augur well for fast, adaptive, and cost-efficient antimicrobial peptide design with programmable physicochemical properties.

## Data Availability

The original contributions presented in the study are included in the article/Supplementary material, further inquiries can be directed to the corresponding author.
