# Supplementary material for: Rational design of potent ultrashort antimicrobial peptides with programmable assembly into nanostructured hydrogels
Source: Front Chem. 2023 Jan 13;10:1009468. doi: 10.3389/fchem.2022.1009468 (PMC9881724; doi:10.3389/fchem.2022.1009468)
Supplement: Supplementary file 1 [file DataSheet1.docx]

Supplementary Material

**Table S1:** Fluorescence activated cell sorting assays of Indolicidin, Pris and P-Pris

| Fluorescence activated cell sorting assay (FACS) | | | | | | | |
| --- | --- | --- | --- | --- | --- | --- | --- |
| **Peptide**  Amino acid sequence | Concentration µM | *e. coli* | | *S. aureus*  *Methicillin*  *resistant* | | *C. auris*  *Fluconazole*  *resistant* | |
|  |  | Live % | Dead % | Live % | Dead % | Live % | Dead % |
| **Indolicidin (control)**  ILPWKWPWWPWRR-NH2 | **42** | 0 | 92 | 30 | 71 | 3 | 97 |
|  | **21** | 1 | 97 | 84 | 19 | 5 | 95 |
|  | **10** | 7 | 89 | 95 | 1 | 58 | 42 |
|  | **5** | 92 | 3 | 93 | 1 | 82 | 15 |
|  | **3** | 94 | 0 | 94 | 1 | 95 | 3 |
| **Priscilicidin**  Fmoc-WWRR-NH2 | **216** | 4 | 77 | 7 | 81 | 4 | 92 |
|  | **108** | 2 | 81 | 1 | 91 | 1 | 93 |
|  | **54** | 10 | 76 | 2 | 93 | 1 | 98 |
|  | **27** | 47 | 44 | 5 | 92 | - | - |
|  | **14** | 94 | 2 | 89 | 3 | 50 | 50 |
| **P-Priscilicidin**  Fmoc-WPWRR-NH2 | **196** | 2 | 89 | 4 | 97 | 2 | 98 |
|  | **98** | 35 | 44 | 19 | 83 | 1 | 98 |
|  | **49** | 33 | 48 | 67 | 56 | 2 | 98 |
|  | **24** | 57 | 33 | 95 | 7 | 4 | 95 |
|  | **12** | 93 | 2 | 95 | 5 | 67 | 33 |

FACS data is shown in the following pages (Figure S2-S25).

| 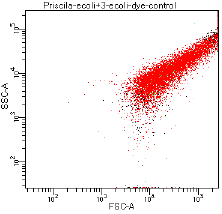 | 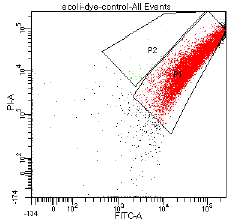 | ***E.coli* Control**  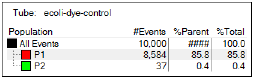 |
| --- | --- | --- |

| 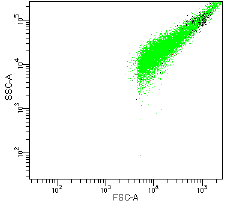 | 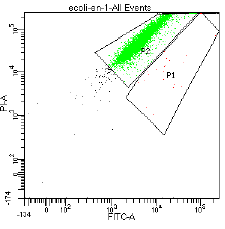 | ***E.coli* + Indolicidin (42** **µM)**  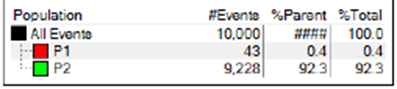 |
| --- | --- | --- |

| 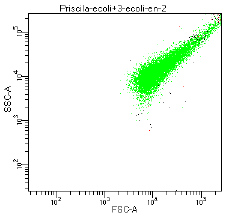 | 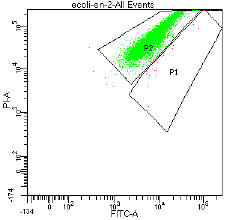 | ***E.coli* + Indolicidin (21** **µM)**  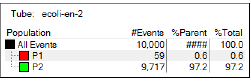 |
| --- | --- | --- |

**Figure S1:** Fluorescence activated cell sorting assay of *E. coli* control and *E. coli* + Indolicidin (42 μM and 21 μM).

| 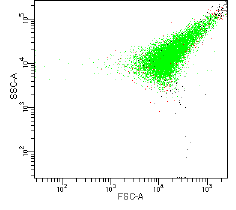 | 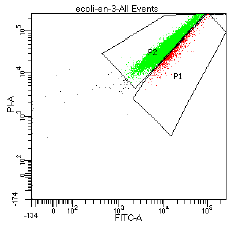 | ***E.coli* + Indolicidin (10** **µM)**  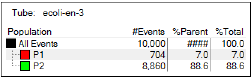 |
| --- | --- | --- |

| 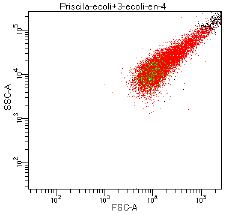 | 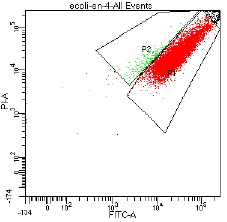 | ***E.coli* + Indolicidin (5** **µM)**  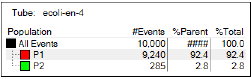 |
| --- | --- | --- |

| 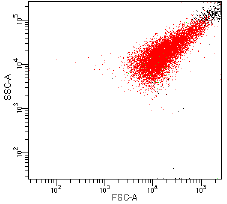 | 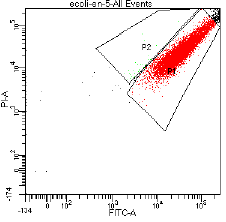 | ***E.coli* + Indolicidin (3** **µM)**  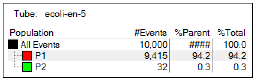 |
| --- | --- | --- |

**Figure S2:** Fluorescence activated cell sorting assay of *E. coli* + Indolicidin (10 μM, 5 μM and 3 μM).

| 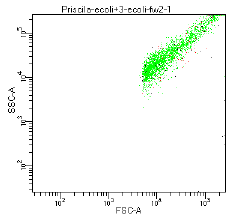 | 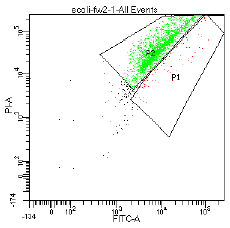 | ***E. coli* + Priscilicidin (216** **µM)**  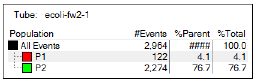 |
| --- | --- | --- |

| 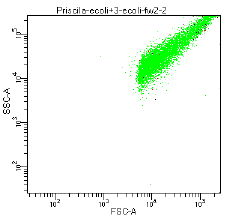 | 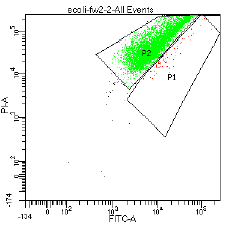 | ***E. coli* + Priscilicidin (108** **µM)**  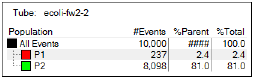 |
| --- | --- | --- |
| 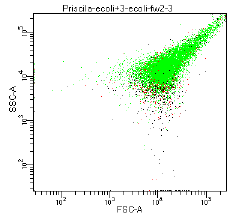 | 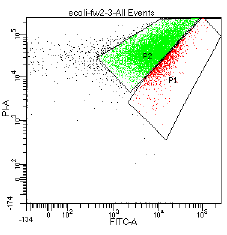 | ***E. coli* + Priscilicidin (54** **µM)**  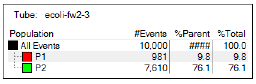 |

**Figure S3:** Fluorescence activated cell sorting assay of *E. coli* + Priscilicidin (216 μM, 108 μM and 54 μM).

| 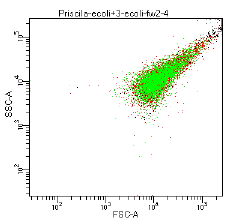 | 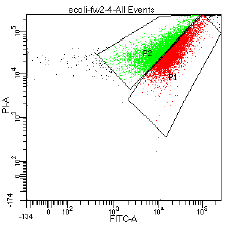 | ***E. coli* + Priscilicidin (27** **µM)**  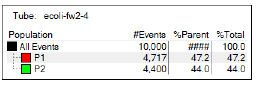 |
| --- | --- | --- |

| 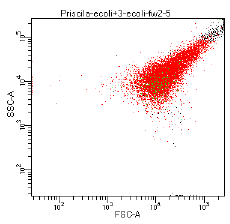 | 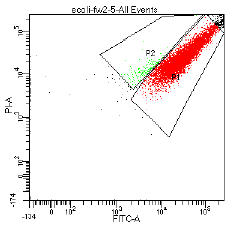 | ***E. coli* + Priscilicidin (14** **µM)**  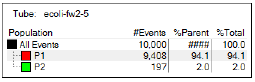 |
| --- | --- | --- |

| 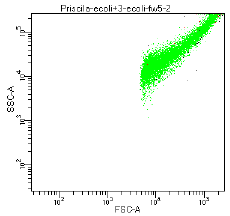 | 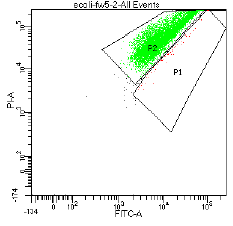 | ***E. coli* + P-Priscilicidin (196** **µM)**  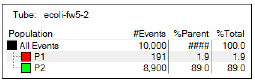 |
| --- | --- | --- |

**Figure S4:** Fluorescence activated cell sorting assay of *E. coli* + Priscilicidin (27 μM and 14 μM) and *E. coli* + P-Priscilicidin (196 μM).

| 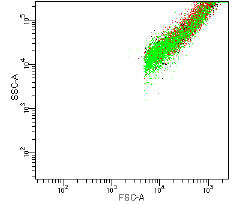 | 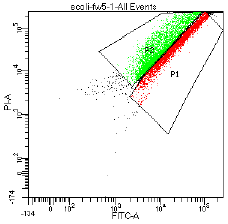 | ***E. coli* + P-Priscilicidin (98** **µM)**  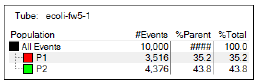 |
| --- | --- | --- |

| 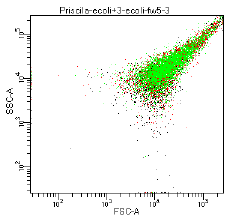 | 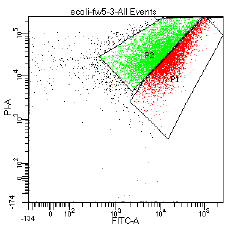 | ***E. coli* + P-Priscilicidin (49** **µM)**  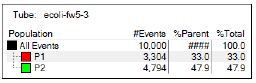 |
| --- | --- | --- |

| 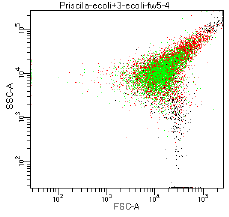 | 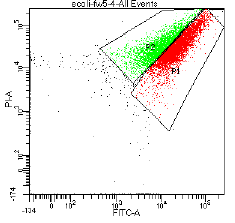 | ***E. coli* + P-Priscilicidin (24** **µM)**  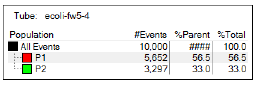 |
| --- | --- | --- |

**Figure S5:** Fluorescence activated cell sorting assay of *E. coli* + P- Priscilicidin (98 μM, 49 μM and 24 μM).

| 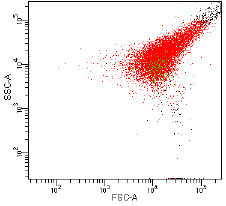 | 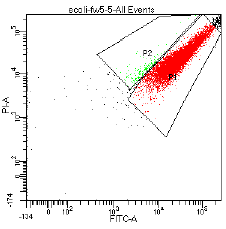 | ***E. coli* + P-Priscilicidin (12** **µM)**  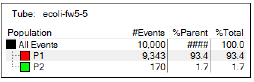 |
| --- | --- | --- |

| 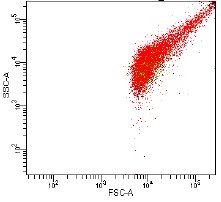 | 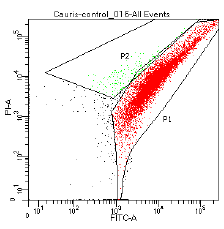 | ***S. aureus (*resistant)**  **Control**  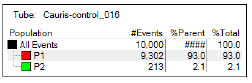 |
| --- | --- | --- |

| 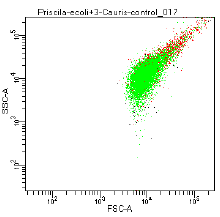 | 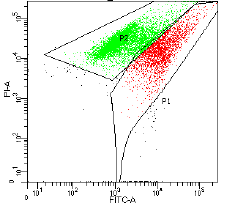 | ***S. aureus (R)* + Indolicidin (42** **µM)**  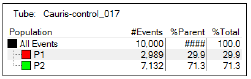 |
| --- | --- | --- |

**Figure S6:** Fluorescence activated cell sorting assay of *E. coli* + P-Priscilicidin (12 μM), *S. aureus (R)* control, and *S. aureus (R)* + Indolicidin (42 μM).

| 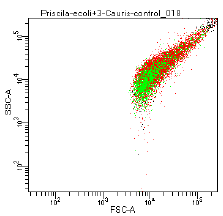 | 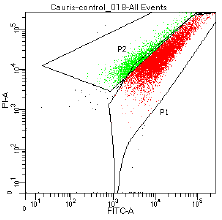 | ***S. aureus(R)* + Indolicidin (21** **µM)**  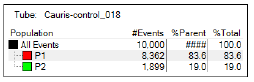 |
| --- | --- | --- |

| 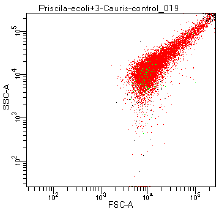 | 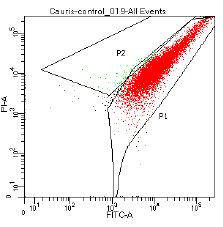 | ***S. aureus(R)* + Indolicidin (10** **µM)**  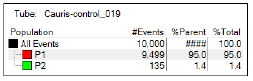 |
| --- | --- | --- |
| 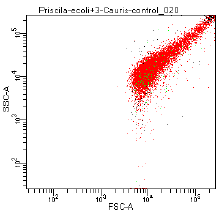 | 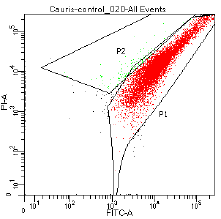 | ***S. aureus(R)* + Indolicidin (5** **µM)**  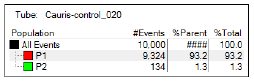 |

**Figure S7:** Fluorescence activated cell sorting assay of *S. aureus (R)* + Indolicidin (21 μM, 10 μM and 5 μM).

| 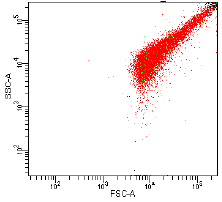 | 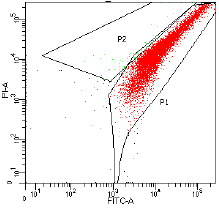 | ***S. aureus(R)* + Indolicidin (3** **µM)**  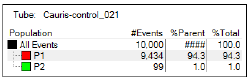 |
| --- | --- | --- |

| 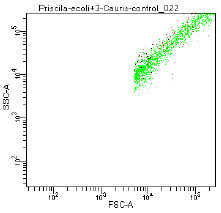 | 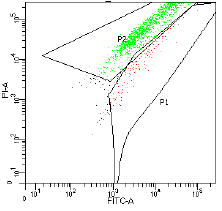 | ***S. aureus(R)* + Priscilicidin (216** **µM)**  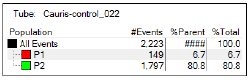 |
| --- | --- | --- |

| 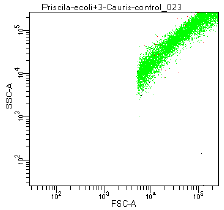 | 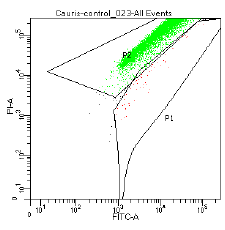 | ***S. aureus(R)* + Priscilicidin (108** **µM)**  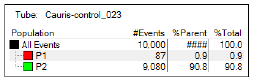 |
| --- | --- | --- |

**Figure S8:** Fluorescence activated cell sorting assay of *S. aureus (R)* + Indolicidin (3 μM) and *S. aureus (R)* + Priscilicidin (216 μM and 108 μM).

| 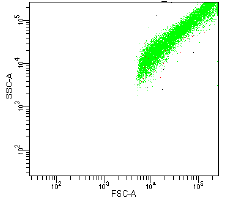 | 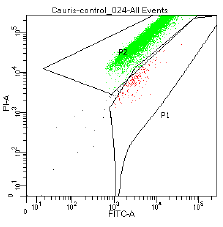 | ***S. aureus(R)* + Priscilicidin (54** **µM)**  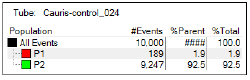 |
| --- | --- | --- |

| 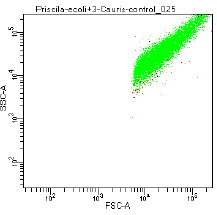 | 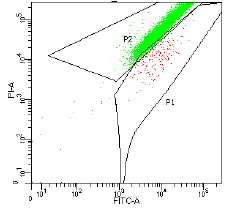 | ***S. aureus(R)* + Priscilicidin (27** **µM)**  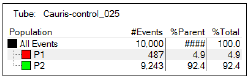 |
| --- | --- | --- |

| 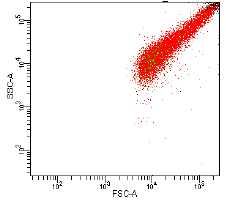 | 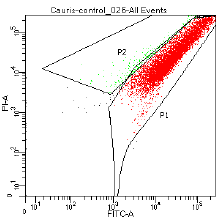 | ***S. aureus(R)* + Priscilicidin (14** **µM)**  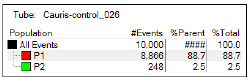 |
| --- | --- | --- |

**Figure S9:** Fluorescence activated cell sorting assay of *S. aureus (R)* + Priscilicidin (54 μM, 27 μM and 14 μM).

| 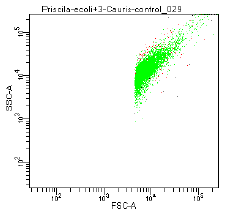 | 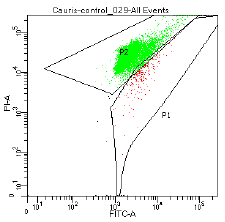 | ***S. aureus(R)* + P-Priscilicidin (196** **µM)**  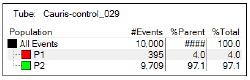 |
| --- | --- | --- |
| 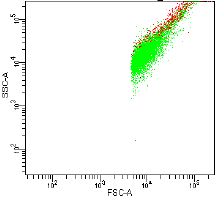 | 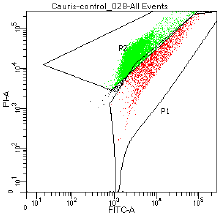 | ***S. aureus(R)* + P-Priscilicidin (98** **µM)**  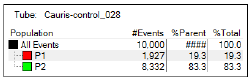 |

| 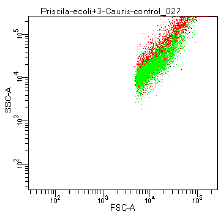 | 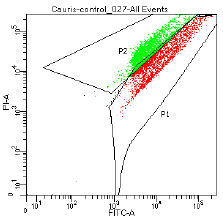 | ***S. aureus(R)* + P-Priscilicidin (49** **µM)**  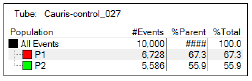 |
| --- | --- | --- |

**Figure S10:** Fluorescence activated cell sorting assay of *S.* *aureus (R)* + P-Priscilicidin (196 μM, 98 μM and 49 μM).

| 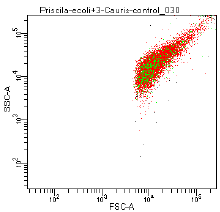 | 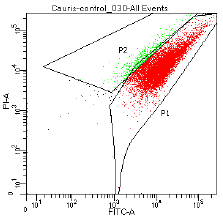 | ***S. aureus(R)* + P-Priscilicidin (24** **µM)**  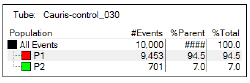 |
| --- | --- | --- |

| 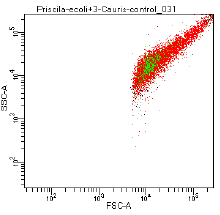 | 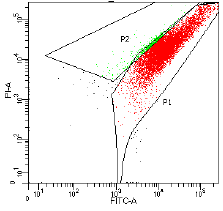 | ***S. aureus(R)* + P-Priscilicidin (12** **µM)**  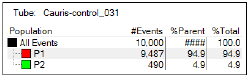 |
| --- | --- | --- |

| 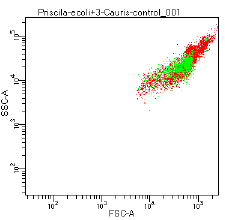 | 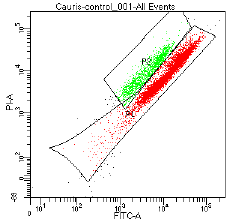 | ***C. auris* (*fluconazole resistant*) Control**  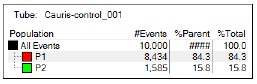 |
| --- | --- | --- |

**Figure S11:** Fluorescence activated cell sorting assay of *S. aureus (R)* + P-Priscilicidin (24 μM and 12) and *C. auris (R)* control.

| 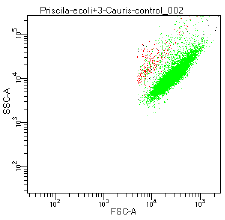 |  | ***C. auris(R)* + Indolicidin (42** **µM)** |
| --- | --- | --- |

|  |  | ***C. auris(R)* + Indolicidin (21** **µM)** |
| --- | --- | --- |

|  |  | ***C. auris(R)* + Indolicidin (10** **µM)** |
| --- | --- | --- |

**Figure S12:** Fluorescence activated cell sorting assay of *C. auris (R)* + Indolicidin (42 μM, 21 μM and 10 μM).

|  |  | ***C. auris(R)* + Indolicidin (5** **µM)** |
| --- | --- | --- |

|  |  | ***C. auris(R)* + Indolicidin (3** **µM)** |
| --- | --- | --- |

|  |  | ***C. auris(R)* + Priscilicidin (216** **µM)** |
| --- | --- | --- |

**Figure S13:** Fluorescence activated cell sorting assay of *C. auris (R)* + Indolicidin (5 μM, 3 μM and 10μM) and *C. auris (R)* + Priscilidin (216 μM).

|  |  | ***C. auris(R)* + Priscilicidin (108** **µM)** |
| --- | --- | --- |
|  |  | ***C. auris(R)* + Priscilicidin (54** **µM)** |

|  |  | ***C. auris(R)* + Priscilicidin (14** **µM)** |
| --- | --- | --- |

**Figure S14:** Fluorescence activated cell sorting assay of *C. auris (R)* + Priscilicidin (108 μM, 54 μM and 14 μM).

|  |  | ***C. auris(R)* + P-Priscilicidin (196** **µM)** |
| --- | --- | --- |

|  |  | ***C. auris(R)* + P-Priscilicidin (98** **µM)** |
| --- | --- | --- |

|  |  | ***C. auris(R)* + P-Priscilicidin (49** **µM)** |
| --- | --- | --- |

**Figure S15:** Fluorescence activated cell sorting assay of *C. auris (R)* + P-Priscilicidin (196 μM, 98 μM and 49 μM).

|  |  | ***C. auris(R)* + P-Priscilicidin (24** **µM)** |
| --- | --- | --- |

|  |  | ***C. auris(R)* + P-Priscilicidin (12** **µM)** |
| --- | --- | --- |

**Figure S16:** Fluorescence activated cell sorting assay of *C. auris (R)* + P-Priscilicidin (24 μM and 12 μM).

|  | | ***Escherichia coli*** | | | | | | |
| --- | --- | --- | --- | --- | --- | --- | --- | --- |
| **Control** | |  |  | | |  | | |
|  |  |  |  | | |  | | |
|  |  |  |  | | |  | | |
|  |  |  | | |  | | | |
|  | **Figure S17.** Assessment of *e. coli* surface morphology performed by SEM (control). | | | | | | |  |
| **Priscilicidin 162 μM** |  | |  | | | |  |  |
|  |  | | |  | | | |  |
| **Priscilicidin 325 μM** |  | |  | | | |  |  |
|  | **Figure S18.** Assessment of *e. coli* surface morphology performed by SEM after the treatment with Pris 162 μM and 325 μM. | | | | | | |  |
| **P-Priscilicidin 147 μM** |  | |  | | | |  |  |
|  |  | | |  | | | |  |
|  |  | | |  | | | |  |
| **P-Priscilicidin 294 μM** |  | |  | | | |  |  |
|  |  | | |  | | | |  |
|  | **Figure S19.** Assessment of *e. coli* surface morphology performed by SEM after the treatment with P-Pris 147 μM and 294 μM.  ***Staphylococcus Aureus (R)*** | | | | | | |  |
| **Control** |  | | |  | | | |  |
|  |  | | |  | | | |  |

**Figure S20.** Assessment of *s. aureus (R)* surface morphology performed by SEM (control).

| **Priscilicidin 81 μM** |  |  | |  |
| --- | --- | --- | --- | --- |
|  |  | |  | |
|  |  | |  | |
| **Priscilicidin 325 μM** |  |  | |  |
|  |  | |  | |

**Figure S21.** Assessment of *s. aureus (R)* surface morphology performed by SEM after the treatment with Pris 81 μM and 325 μM.

| **P-Priscilicidin 73 μM** |  |  | |  |
| --- | --- | --- | --- | --- |
|  |  | |  | |
|  |  | |  | |
| **P-Priscilicidin 145 μM** |  |  | |  |
|  |  | |  | |

**Figure S22.** Assessment of *s. aureus (R)* surface morphology performed by SEM after the treatment with P-Pris 73 μM and 145 μM.

|  | ***Candida Auris (R)*** | |
| --- | --- | --- |
| **Control** |  |  |
|  |  |  |

**Figure S23.** Assessment of *c. auris (R)* surface morphology performed by SEM (control).

| **Priscilicidin 41 μM** |  | |  | |
| --- | --- | --- | --- | --- |
|  |  | |  | |
| **Priscilicidin 81 μM** |  |  | |  |
|  |  | |  | |

**Figure S24.** Assessment of *c. auris (R)* surface morphology performed by SEM after the treatment with Pris 41 μM and 81 μM.

| **P-Priscilicidin 37 μM** |  |  |
| --- | --- | --- |
|  |  |  |
| **P-Priscilicidin 73 μM** |  |  |

**Figure S25.** Assessment of *c. auris (R)* surface morphology performed by SEM after the treatment with P-Pris 37 μM and 73 μM.

1. **Priscilicidin**

1. **P-Priscilicidin**

**Figure S26.** ATR-FTIR shifting vibrations in the amide I region (1700-1600 cm-1) for Priscilicidin and P-Priscilicidin in water at 5 and 10% w/w at different time points.

**Figure S27.** Cryogenic transmission electron microscopy imaging of a) 5% w/w Priscilicidin acetate in water, 1 day incubation at 5°C; b) 10% w/w Priscilicidin acetate in water, 1 day incubation at 5°C.

**Figure S28.** Synchrotron small angle X-ray scattering performed for [1-10]% wt Priscilicidin acetate in water, recorded at 25°C, for incubation times of respectively 3 days (A), 10 days (B) and 1 month (C).

**Figure S29.** Synchrotron small angle X-ray scattering performed for [1-10]% wt Priscilicidin acetate in water, recorded at 37°C, for incubation times of respectively 3 days (A), 10 days (B) and one month (C).

**Figure S30.** Synchrotron small angle X-ray scattering performed for [1-10]% wt P-Priscilicidin acetate in water, recorded at 25°C, for incubation times of respectively 0.5 day (A), 10 days (B) and one month (C).

**Figure S31.** Synchrotron small angle X-ray scattering performed for [1-10]% wt P-Priscilicidin acetate in water, recorded at 37°C, for incubation times of respectively 0.5 day (A), 10 days (B) and one month (C).

**Figure S32.** Oscillatory rheology: frequency sweep tests for Priscilicidin acetate [1-10]% wt in water at different times of incubation, respectively 0.5 day (A), 5 days (B) and 1 month (C).
